# Neonatal Diabetes Mellitus Accompanied by Diabetic Ketoacidosis and Mimicking Neonatal Sepsis: A Case Report

**DOI:** 10.4274/jcrpe.v2i3.131

**Published:** 2010-08-08

**Authors:** Ayhan Abacı, Cem Hasan Razi, Osman Özdemir, Samil Hızlı, Fatih Kıslal, Pınar Işık Argas, Nimet Kabakuş

**Affiliations:** 1 Kecioren Training Research Hospital, Pediatric Endocrinology, Ankara, Turkey; 2 Kecioren Training and Research Hospital, Pediatrics, Ankara, Turkey; 3 Kecioren Training and Research Hospital, Pediatric Gastroenterology, Hepatology and Nutrition, Ankara, Turkey; 4 Kecioren Training and Research Hospital, Pediatric Cardiology, Ankara, Turkey; +90 312 356 90 00+90 312 356 90 02ayhanabaci@gmail.comDepartment of Pediatric Endocrinology, Keciören Training and Research Hospital, Ankara, Turkey

**Keywords:** Neonate, diabetes mellitus, ketoacidosis

## Abstract

Neonatal diabetes mellitus (DM) develops within the first six weeks of life with basic findings including dehydration, hyperglycaemia, and mild or no ketonemia/ketonuria. It can be either transient or permanent. Here, we report a case of a one−month−old infant with permanent neonatal diabetes, due to pancreatic hypoplasia, accompanied by diabetic ketoacidosis (DKA). The hyperglycaemia and ketoacidosis resolved by the 14^th^ hour of treatment, consisting of IV insulin and rehydration. Subsequently, insulin treatment was continued with neutral protamine hagedorn (NPH) insulin. Breastfeeding was started and was continued at intervals of three hours. Following initiation of breastfeeding, the stools became watery, loose, yellow−green in color, and frequent (8−10 times a day). They contained no blood or mucus. Replacement of pancreatic enzymes resulted in decreased stool frequency. Neonatal DM due to pancreatic hypoplasia and associated with DKA may mimic sepsis and should be kept in mind in all newborns who present with fever, dehydration, and weight loss.

**Conflict of interest:**None declared.

## INTRODUCTION

Diabetes mellitus (DM) is a heterogeneous group of metabolic diseases and can present at any age, from birth to old age. Transient and permanent neonatal diabetes mellitus (TNDM and PNDM), which develop within the first few weeks or months of life, are very rare conditions with an incidence of 1 in 300,000 to 500,000 live births (1, 2). TNDM is a developmental disorder of insulin production that resolves in the postnatal period and is mostly associated with intrauterine growth retardation (2). 50−60% of cases of neonatal diabetes are TNDM cases, and they recover within a year. PNDM is less common than the transient form and, in these patients, insulin dependence is lifelong. Hyperglycaemia, failure to thrive, and dehydration occur after birth (1). Insulin production is inadequate, requiring exogenous insulin therapy (3). Here, we report a rare case of a patient with PNDM accompanied by diabetic ketoacidosis (DKA), who was admitted to the neonatal intensive care unit (NICU) with suspicion of neonatal sepsis.

## CASE REPORT

**Figure 1:**
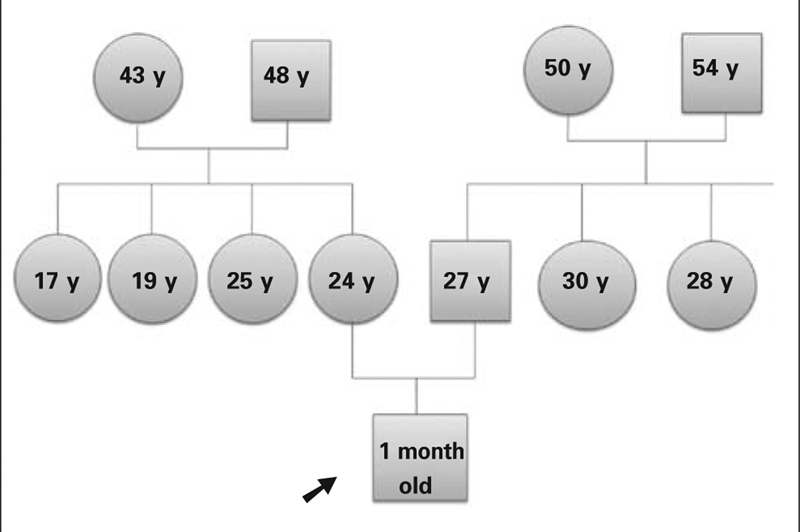
Pedigree of the patient’s family

A one−month−old boy was referred to the NICU at Kecioren Training and Research Hospital, Ankara, because of fever, lethargy, and poor feeding. He was born healthy at 40 gestational weeks following an uncomplicated pregnancy. The birth weight was in the lower limit of the normal range for gestational age (2550 g) and the newborn did not have any dysmorphic abnormalities at birth. Breastfeeding was started shortly after delivery. However, frequent stools were not observed by the family until the patient was admitted to our hospital. He was the first child of unrelated parents. There was no family history of diabetes or autoimmune diseases (Figure 1).

On physical examination, the infant was irritable, drowsy, approximately 8−10% dehydrated (as estimated by a sunken fontanelle, sunken eyes and reduced skin turgor), and tachycardic (176 beats/min). His respiratory rate was 64 breaths/min, body temperature was 39.1°C (rectal), and blood pressure was 65/45 mmHg. The source of the fever was not apparent. First−line laboratory analyses revealed the following results: Blood chemistry: glucose: 1125 mg/dL, Na: 129 mmol/L, K: 5.1 mmol/L, Cl: 94 mmol/L, Ca: 10.8 mg/dL, (venous blood gas analysis), pH: 7.36, HCO3: 5 mEq/L, and pCO_2_: 9 mmHg; Urinalysis: specific gravity 1.028, glucose 4+, ketones 3+. Serum insulin level was 0.9 μU/mL and C−peptide 0.5 ng/mL, while blood sugar was 1132 mg/dL at this time. The glycosylated hemoglobin level (high−pressure liquid chromatography method, HPLC) was high (10.6%). Anti−insulin, anti−islet cell and anti−glutamic acid decarboxylase antibodies were found to be negative. Fecal fat was negative. Thyroid function tests (TSH: 2.1 μU/mL, fT4: 1.02 ng/dL) were within normal ranges. Cranial ultrasonography and computed tomography were normal. Imaging study of the abdomen (i.e. magnetic resonance imaging) showed hypoplastic pancreatic tissue. Echocardiography revealed no abnormality. The parents' fasting blood glucose levels were normal.

A diagnosis of DKA was made and treatment was started with intravenous insulin (0.05 IU/kg/hour for 2 hours and 0.1 IU/kg/hour thereafter) and a rehydration solution. Insulin and glucose concentrations were subsequently adjusted according to the rate of glucose fall, and no complications were observed. Hyperglycaemia and ketoacidosis resolved by the 14^th^ hour of treatment. Breastfeeding was resumed with feedings at three−hour intervals. Insulin treatment was continued with subcutaneous neutral protamine hagedorn (NPH) (0.8 IU/kg/day in 4 doses) insulin. Following the start of breastfeeding, we observed watery, loose, yellow−green colored, and frequent (8−10 times a day) stools, which contained no blood or mucus. Although fecal fat was negative, pancreatic enzyme replacement therapy was initiated due to the frequent stools and finding of pancreatic hypoplasia on imaging studies.Replacement of pancreatic enzymes (40 droplets/HPF) resulted in decreased stool frequency (2−4 times a day).

The infant was discharged 3 weeks after admission. During the follow−up, insulin dose was decreased (from 4 doses of NPH to 2 doses of NPH), but increased again, due to increment in blood glucose levels to 200−350 mg/dL, suggesting a diagnosis of PNDM.

Currently, the patient is ten months old and is on two doses of NPH (0.25 IU/kg/day). His most recent HbA1c level was 7.4%.

## DISCUSSION

PNDM is less common than TNDM and never goes into remission (2). It accounts for approximately 40−50% of neonatal DM cases (4). There are no clinical features that can predict whether a neonate with diabetes without any dysmorphology will eventually have PNDM or TNDM (2). Metz et al (5) showed that PNDM was less likely to be associated with intrauterine growth restriction compared to TNDM. However, severe intrauterine growth retardation has been reported in PNDM associated with pancreatic agenesis (6). Our case had pancreatic hypoplasia, and his birth weight was on the lower limit of normal range.

The etiology of neonatal DM is unclear and its pathogenesis differs from insulin−dependent childhood DM. Presence of islet cell antibodies has not been reported in neonatal DM (7). Anti−insulin, anti−islet cell and anti−glutamic acid decarboxylase antibodies were also negative in our patient.

A number of syndromic and genetic constellations, such as Wolcott−Rallison syndrome (autosomal recessive), immune dysregulation, polyendocrinopathy, enteropathy, X−linked (IPEX) syndrome (X−linked, recessive), phosphoribosyl−ATP pyrophosphate hyperactivity (X−linked), and glucokinase deficiency (MODY2) have been reported in PNDM (2, 4). Wolcott−Rallison syndrome is characterized by the appearance of PNDM within the first few weeks of life. It is also related with multiple epiphyseal or spondylo−epiphyseal dysplasia, cardiac anomaly, recurrent hepatitis, and ectodermal dysplasia (1, 4, 8). The other associated syndrome is IPEX, the major clinical signs of which are early−onset insulin−dependent DM, chronic diarrhea with villous atrophy, eczema, anaemia, thrombocytopenia, severe enteropathy, and eventually other endocrinopathies (9). In our patient, there were no signs of the above−mentioned abnormalities.

Pancreatic agenesis/hypoplasia is a clinical entity, which develops due to mutations in insulin promoter factor−1 (IPF−1) gene and pancreas transcription factor 1 alpha (PTF1A) (1). It may be associated with other abnormalities such as cerebellar hypoplasia, cardiac septal defects or absence of the gallbladder (10, 11). Investigations for presence of other abnormalities in our patient revealed normal results. In addition, pancreatic agenesis/hypoplasia is characterized with early−onset PNDM with no ketoacidosis or signs of malabsorption due to pancreatic exocrine dysfunction. In our case, we have observed pancreatic hypoplasia on imaging studies. Frequent stools following breastfeeding also suggested an associated pancreatic exocrine deficiency. Frequent stools may not be observed immediately after birth and may be delayed up to one month of age (12), as seen in our patient.

It was reported that especially in TNDM, ketonuria is usually mild or absent; but DKA may develop when the diagnosis is delayed (1, 4, 6, 13). Wooley et al (4) emphasized in a case report that these children may present to emergency departments with a picture mimicking sepsis, just like in our case. We think that the cause of DKA in our case was due to delayed diagnosis or late admission to the emergency unit.

Insulin therapy is crucial in neonatal DM to obtain satisfactory weight gain and growth in these infants. A variety of methods for providing insulin such as: intravenous infusion, short−acting and long−acting subcutaneous injections, or continuous subcutaneous insulin infusion (CSII) can be used (4). In a study by Mitamura et al (14), control of the blood glucose concentration in infants with TNDM was attained with ultralente insulin treatment without any episodes of hypoglycaemia. These authors recommended subcutaneous injection of ultralente insulin, rather than lente or isophane (NPH) insulin to avoid hypoglycaemia during treatment. In recent publications, although there is currently no licence for its use in this age group, insulin glargine treatment is suggested, because of its flat pharmacokinetic profile which might prove useful in this condition (15). In some centres in Europe, the use of CSII in all cases of neonatal DM is proposed, stating that during the neonatal period, CSII therapy is safe, more physiological, more accurate and easier to manage than injections (16). Due to the existing controversies about the treatment option, most cases of neonatal DM were treated with NPH (4, 6, 13).In our case, blood glucose levels were successfully controlled with 2−4 daily doses of NPH insulin treatment, and no serious complications have been observed.

In conclusion, pancreatic hypoplasia is a rare cause of neonatal DM and is not always associated with intrauterine growth retardation. Also, neonatal DM due to pancreatic hypoplasia and associated with DKA may mimic sepsis and should be kept in mind in all newborns who present with fever, dehydration, and weight loss. These patients can be successfully treated with NPH given in 2−4 doses per day.
